# Evaluation of Intermittent Hemodialysis in Critically Ill Cancer Patients with Acute Kidney Injury Using Single-Pass Batch Equipment

**DOI:** 10.1371/journal.pone.0149706

**Published:** 2016-03-03

**Authors:** Verônica Torres da Costa e Silva, Elerson C. Costalonga, Ana Paula Leandro Oliveira, James Hung, Renato Antunes Caires, Ludhmila Abrahão Hajjar, Julia T. Fukushima, Cilene Muniz Soares, Juliana Silva Bezerra, Luciane Oikawa, Luis Yu, Emmanuel A. Burdmann

**Affiliations:** 1 Nephrology Division, Sao Paulo State Cancer Institute, University of Sao Paulo Medical School, Sao Paulo, Sao Paulo, Brazil; 2 Intensive Care Unit Department, Sao Paulo State Cancer Institute, University of Sao Paulo School Medical School, Sao Paulo, Sao Paulo, Brazil; 3 LIM 12, Division of Nephrology, University of Sao Paulo Medical School, Sao Paulo, Brazil; Sao Paulo State University, BRAZIL

## Abstract

**Background:**

Data on renal replacement therapy (RRT) in cancer patients with acute kidney injury (AKI) in the intensive care unit (ICU) is scarce. The aim of this study was to assess the safety and the adequacy of intermittent hemodialysis (IHD) in critically ill cancer patients with AKI.

**Methods and Findings:**

In this observational prospective cohort study, 149 ICU cancer patients with AKI were treated with 448 single-pass batch IHD procedures and evaluated from June 2010 to June 2012. Primary outcomes were IHD complications (hypotension and clotting) and adequacy. A multiple logistic regression was performed in order to identify factors associated with IHD complications (hypotension and clotting). Patients were 62.2 ± 14.3 years old, 86.6% had a solid cancer, sepsis was the main AKI cause (51%) and in-hospital mortality was 59.7%. RRT session time was 240 (180–300) min, blood/dialysate flow was 250 (200–300) mL/min and UF was 1000 (0–2000) ml. Hypotension occurred in 25% of the sessions. Independent risk factors (RF) for hypotension were dialysate conductivity (each ms/cm, OR 0.81, CI 0.69–0.95), initial mean arterial pressure (each 10 mmHg, OR 0.49, CI 0.40–0.61) and SOFA score (OR 1.16, CI 1.03–1.30). Clotting and malfunctioning catheters (MC) occurred in 23.8% and 29.2% of the procedures, respectively. Independent RF for clotting were heparin use (OR 0.57, CI 0.33–0.99), MC (OR 3.59, CI 2.24–5.77) and RRT system pressure increase over 25% (OR 2.15, CI 1.61–4.17). Post RRT blood tests were urea 71 (49–104) mg/dL, creatinine 2.71 (2.10–3.8) mg/dL, bicarbonate 24.1 (22.5–25.5) mEq/L and K 3.8 (3.5–4.1) mEq/L.

**Conclusion:**

IHD for critically ill patients with cancer and AKI offered acceptable hemodynamic stability and provided adequate metabolic control.

## Introduction

An increasing number of patients with cancer have been admitted to intensive care units (ICU) worldwide, accounting for up to 15% of all ICU admissions [1, 2]. The survival of these patients has increased in the last decades [3–6] and approximately up to 49% of them experience an episode of acute kidney injury (AKI) during the ICU stay; 9% to 32% of them require renal replacement (RRT) [7–9].

The few reports assessing RRT in cancer patients with AKI in the ICU focused on survival prognostic factors [5, 6, 10–12] and included mostly hematologic cancer patients [5, 10–12]. Data about the technical characteristics of RRT in these patients, such as modality, RRT dose, anticoagulation, complications and efficiency are notably scarce, especially in patients with solid tumors. In addition, cancer patients have peculiar characteristics, such as release of pro-inflammatory cytokines and a pro-thrombotic status that may impact on hemodynamic stability and clotting during RRT sessions.

Intermittent hemodialysis (IHD) is widely used for AKI patients in the critical care setting [13, 14]. However, there is no data assessing the safety and efficiency of IHD in cancer patients with AKI in the ICU. The aim of this study was to assess the complications and adequacy of IHD in ICU cancer patients with AKI admitted to the ICU of a tertiary Brazilian cancer hospital.

## Methods

This is a prospective cohort of critically ill cancer patients with AKI who were treated with RRT in the ICU at the Instituto do Câncer do Estado de São (ICESP), Faculdade de Medicina da Universidade de São Paulo, a tertiary academic hospital in Brazil, exclusively devoted to the care of cancer patients. This unit is an open access ICU with 71 beds, and admits approximately 3,000 patients yearly. All admitted patients are candidates to “ICU trial” strategy (full treatment procedures with latter reassessment). The study was approved by the local ethics committee (Coordinating body of Ethics in Research, number 164/13). The institutional review board waived the need for consent due to the observational nature of the study. The study was performed in compliance with the ethical standard of Brazilian laws.

### Patients

All AKI patients admitted to the ICU from June, 2010 to June, 2012 receiving RRT at any time during their ICU stay were evaluated. Only AKI patients who received IHD were included for analysis. They were prospectively followed from RRT initiation until hospital discharge or death. AKI was diagnosed according to the AKIN criteria [15]. CKD (chronic kidney disease) was defined as two estimated glomerular filtration rate (eGFR) < 60 ml/min/1.73 m2 in an interval ≥ three months [16]. Baseline serum creatinine (SCr) was considered as the mean of all values obtained in the last three months before hospital admission or the minimum value achieved during hospitalization. Sepsis was defined according to established criteria [17]. The exclusion criteria were baseline SCr ≥ 3.5 mg/dL, CKD on maintenance RRT, age < 18 years old, kidney transplantation and dysnatremia (serum sodium 10 mEq/L above or below 138 mEq/L) that could not be managed by the standard dialysate used in the single-pass batch (SPB) hemodialysis equipment.

Data were collected by the nephrology team responsible for RRT. Daily visits were performed until renal recovery, hospital discharge or death. The data collected included age, gender, cancer related variables (tumor type and extension, previous treatment, disease status), co-morbidities, hospital and ICU length of stay, baseline SCr and AKI-related factors (sepsis, nephrotoxicity, urinary obstruction, surgery). On the day of IHD, blood pressure, weight, urinary output, need for vasoactive drugs and the use of mechanical ventilation were recorded. Illness severity was assessed by the Sequential Organ Failure Assessment (SOFA) score [18].

### IHD procedures

Decisions regarding the need for RRT (hyperkalemia, acidosis, urea above 180 mg/dL, and fluid overload with pulmonary edema), timing and modality (IHD or continuous RRT) are made in agreement by the nephrology and ICU teams. CRRT was indicated for patients with two or more organ failures or receiving vasoactive drugs (noradrenaline > 0.2 mcg/Kg/min and/or dobutamine > 5 mcg/Kg/min). IHD was preferably initiated in hemodynamically stable patients, i.e. those with no vasoactive drugs or using only at low levels (noradrenaline < 0.2 mcg/Kg/min and/or dobutamine < 5 mcg/Kg/min).

All IHD procedures were performed with SPB equipment (Genius, Fresenius Medical Care, Germany), consisting of an air-free glass dialysate container of 90 L of ultrapure dialysate (batch-system) connected to a bicameral roller pump capable of simultaneously and equally pumping blood and dialysate (in this system blood flow is always equal to dialysate flow). IHD procedures were prescribed by the nephrology team and managed by nephrology specialized nurses. Two types of polysulfone capillaries (Fresenius medical care, Germany) were used: FX60 (surface area 1.4m2) and FX80 (surface area 1.8m2). Dialysate composition was sodium 138 mEq/L, bicarbonate 35 mEq/L, magnesium 1.0 mEq/L and chloride 110.5 mEq/L. Calcium (2.5–3.5 mEq/L) and potassium (2.0–3.0 mEq/L) were individualized according to the patients´ conditions. SPB does not allow a sodium dialysate profile. Final dialysate conductivity is mainly determined by sodium dialysate, presenting mild variances depending on the dialysate composition and temperature [19]. The dialysate is heated at two temperature levels, medium (approximately 38°C) or low (approximately 37°C), and spontaneous cooling occurs (0.3°C/hour) in the following hours [20]. Two nephrology nurse shifts were responsible for RRT initiation (1st: from 7:00 AM to 7:00 PM; 2nd: from 7:00 PM to 7:00 AM). IHD included both sustained low efficiency dialysis (SLED) and conventional hemodialysis (CHD) modalities, defined by dialysis duration up to four and six hours, respectively. Blood/dialysate flow was chosen according to nephrologist discretion depending on patients’ characteristics and daily exams. Anticoagulation was performed using non-fractionated heparin or continuous 0.9% saline flush (saline flow at mL/h equal to blood flow in mL/min). All filters used were flushed before the initiation of RRT with a solution of one liter of 0.9% saline plus 5,000 IU of non-fractionated heparin, followed by one liter of 0.9% saline. Vascular access was performed using temporary catheters of 11.5F length 16 or 20 cm, (Arrow International, USA). SPB has a single system pressure reflecting the whole isolated circuit, which was monitored during all RRT sessions.

### Complications

Hypotension was defined as a mean arterial pressure ≤ 65 mmHg and/or the need to introduce or increase vasoactive drugs during the IHD session. We performed sensitivity analysis testing model performances including hypotension definition of MAP < 75 and < 70 mmHg, but the adopted cut-off of MAP < 65 mmHg provided the most consistent model with a better calibration performance (Hosmer-Lemeshow) and accuracy (ROC curve)(data not shown).

The presence of arrhythmias, seizures, need for mechanical ventilation and stroke during IHD were also recorded. Major bleeding was defined as overt bleeding leading to either hypotension or transfusion ≥ two packed red cell units occurring during the day of IHD or the next 48 h [21]. A malfunctioning catheter (MC) was defined as line reversion or reduction of prescribed blood flow during the procedure, and clotting was defined as the coagulation of lines and/or filters resulting in IHD interruption.

### Laboratory parameters and adequacy

Daily blood laboratory tests (hemogram, platelets, biochemistry, blood gases, coagulation profile, C reactive protein, and lactate) were assessed. Urea, creatinine, potassium, sodium, bicarbonate and phosphate were assessed immediately before and after each IHD session. The urea reduction rate and single pool Kt/V were calculated for each IHD session [22]. These parameters were used to assess IHD adequacy. All of the exams performed were part of the ICU and nephrology routine assistance. No further exams were collected for the purpose of this study.

### Outcomes

The primary outcomes were complication rates (hypotension and clotting) and adequacy during IHD procedures. Secondary outcomes were hospital mortality, end-of- life care decision, hospital and ICU length of stay.

### Statistical analysis

All continuous variables were tested for normal distribution using the Kolmogorov-Smirnov test. Data are expressed as mean ± SD or median with 25th and 75th interquartile ranges (IQR), as appropriate and compared using unpaired Student´s t-test (normally distributed data) or the Mann-Whitney U test (non-normally distributed data). Categorical variables are expressed as proportions and were analyzed by Pearson’s χ2 test for independent groups. The Fisher’s test was used where appropriate. Multiple logistic regression models were constructed with backwards variable selection using a *P*-value < 0.05 for variable retention. At the logistic regression model for hypotension, we also tested models with *P* value < 0.1 (data not shown) and the model depicted on the manuscript was the one demonstrating the best calibration and discrimination. We also used mixed effect for repeated measures logistic regression models in order to reduce possible bias. Candidate variables were those with a likelihood ratio of significance < 0.05 upon univariate analysis, which was performed based on terms of comparative groups (with and without outcomes) and not on repeated measures analysis. The colinearity of the maximal models was evaluated using the criteria proposed by Belsley. Discrimination was assessed using the area under the receiver operating characteristic curve [23]. Calibration was assessed using the Hosmer-Lemeshow goodness-of-fit test comparing observed vs. expected mortality across deciles of risk [24]. A high *P*-value (> 0.05) indicated a good fit of the model. A two-tailed *P*-value < 0.05 was considered significant. Statistical analysis was carried out using SPSS for Windows, version 18.0 (Chicago, IL, USA).

## Results

**Fig 1:**
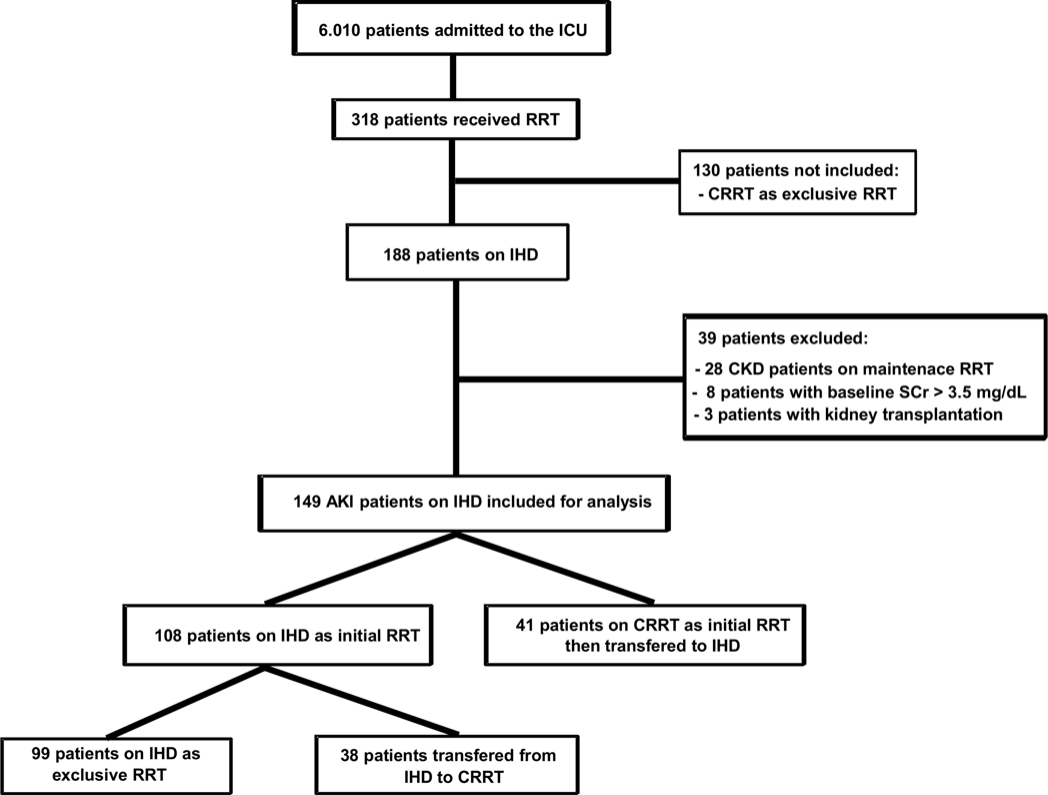
Flowchart of study population. ICU: intensive care unit; RRT, renal replacement therapy; CRRT, continuous renal replacement therapy; IHD, intermittent hemodialysis; CKD, chronic kidney disease; AKI: acute kidney injury, SCr, serum creatinine.

During the study period, 6,010 cancer patients were admitted to the ICU. Among them, 318 patients received RRT (5.29%), 130 were treated exclusively with continuous renal replacement therapy (CRRT) and 188 patients were treated with IHD during their ICU stay. The final analyzed sample was comprised of 448 IHD sessions performed on 149 AKI patients who fulfilled the eligibility criteria (Fig 1). IHD was the initial RRT modality in 108 (72.5%) patients. The other 27.5% were transferred to IHD after 4.0 (2.0–7.0) days of CRRT. Thirty eight (25.5%) patients were transferred from IHD to CRRT due to hemodynamic instability. Fifty (34.1%) of the patients received both IHD and CRRT modalities during their ICU stay.

### Patients and IHD characteristics

Patients were 62.2 ± 14.3 years old, 67.8% were male, 86.6% had a solid tumor and 78.5% had uncontrolled cancer. The baseline SCr was 1.0 (0.86–1.70) mg/dL, baseline eGFR was 66.7 (41.9–89.4) ml/min and 42.6% of patients had CKD. Other co- morbidities were hypertension (55%), diabetes (22.1%) and cardiovascular disease (26.9%). The main AKI etiologic factors were sepsis (51%), obstructive uropathy (27.5%) and postoperative (24.2%). At the first IHD session, the patients’ SOFA score (without renal points) was 4.0 (2.0–5.75), 8.7% of the patients were on mechanical ventilation and 10.1% were receiving vasoactive drugs (Table 1). Hospital mortality was 59.7%, and the main causes of death were sepsis/multiple organ dysfunction (68.5%) and cancer progression (14.6%). A total of 54 surviving patients (90%, 54/60) were dialysis independent at hospital discharge.

**Table 1:** Parameters of the 149 critically ill cancer patients with AKI treated by intermittent hemodialysis.

Parameters	
Age (years)	62.2 ± 14.3
Male gender	101 (67.8%)
Weight (Kg)	67.0 (64.0–77.7)
Primary cancer	
Urinary	69 (46.3%)
Gynecological	17 (11.4%)
Gastrointestinal	20 (13.4%)
Lymphoma/leukemia/myeloma	20 (13.4%)
Other*	23 (15.5%)
Metastatic tumor (for solid cancer)	54 (36.2%)
Cancer therapy	
Chemotherapy	64 (43%)
Surgery	75 (50.3%)
Radiation	32 (21.5%)
Cancer status	
No tumor evidence	9 (6%)
Controlled/stable on treatment	23 (15.4%)
Uncontrolled newly diagnosed/waiting for treatment	48 (32.2%)
Uncontrolled recurrence/progression	69 (46.3%)
Main Comorbidities	
CKD (Stage III or above)	59 (42.6%)
Hypertension	82 (55%)
Diabetes mellitus	33 (22.1%)
Heart failure/Coronary disease/Vascular disease	40 (26.9%)
Hospital LOS prior to ICU admission (days)	1.00 (0.0–4.50)
Pre ICU patient origin	
Emergency room	58 (38.9%)
Ward	52 (34.9%)
Surgical center	28 (18.8%)
AKI related factors	
Sepsis	76 (51%)
Surgery	36 (24.2%)
Obstructive uropathy	41 (27.5%)
Chemotherapy nephrotoxicity†	10 (6.7%)
Baseline serum creatinine (mg/dL)	1.0 (0.86–1.70)
Variables on first IHD day	
SOFA score (without renal points)	4.0 (2.0–5.75)
Mechanical ventilation	13 (8.7%)
Vasoactive drugs	15 (10.1%)
Diuresis (mL/24 h)	320 (50–1062)
Serum creatinine (mg/dL)**	5.75 (4.0–7.6)
Serum urea (mg/dL)**	190 (130 –m232)
Serum bicarbonate (mEq/L)**	20.7 (18.2–24.8)
Serum potassium (mEq/L)**	4.32 (3.7–4.9)
Outcome	
ICU LOS (days)	10.0 (5.0–18.0)
Hospital LOS (days)	21.0 (13.0–35.0)
End-of-life care decision	24 (16.1%)
Hospital mortality	89 (59.7%)

Results are expressed in number (%), mean ± SD or median (25–75 IQR). SD, standard deviation; IQR, interquartile range; CKD, chronic kidney disease; LOS, length of stay; ICU, intensive care unit; AKI, acute kidney injury; SOFA, sequential organ failure assessment.

*Other primary cancers: lung, breast, head and neck, skin, brain, bone tumors

†Nephrotoxic chemotherapy (platines, gemcitabine, asparaginase, methotrexate) on the last four weeks before ICU admission

** Routine ICU daily exams.

IHD was initiated 4.0 (1.0–7.0) days after ICU admission, and each patient received 2.0 (1.0–3.0) IHD sessions in 2.5 (1.0–7.0) days of therapy. Eighty nine patients (63.8%) received up to two sessions, 34 (22.8%) patients received three to four sessions and only 20 (13.4%) patients performed five or more sessions. IHD session time was 240 (180–300) min, blood/dialysate flow was 250 (200–300) mL/min and the prescribed UF was 1000 (0–2000) ml. The prescribed session time was totally achieved in 75.7% of procedures, and the prescribed UF was totally achieved in 70.3% of the procedures.

Final dialysate conductivity was 139 (138–140) ms/cm, ranging from 138 to 142 ms/cm (Table 2). Considering middle session versus initial system pressure, we observed an increase of 9.5% (0–26.6) during IHD, and 25.2% of the sessions had an increase over 25%. Saline flushing was used as anticoagulation in 314 sessions (70.1%). When heparin was used, the dose was 45 (37.3–74.6) IU/Kg. The most frequent contra- indications for heparin use are depicted in Table 2.

**Table 2:** Parameters of intermittent hemodialysis sessions (n = 448).

Variables	
Blood/Dialysate flow (ml/min)	250 (200–300)
Dialysis duration (min)	240 (180–300)
Dialysis duration ≥ 5 h	153 (27.5%)
Number of sessions per patient	2.0 (1.0–3.0)
Membrane filter FX80	316 (70.6%)
Prescribed UF (mL/session)	1000 (0.00–2000)
Dialysate conductivity (mEq/L)	139 (138–140)
System pressure	
Initial	150 (105–200)
Middle	170 (140–220)
Final	200 (150–250)
System pressure increase (initial vs. middle)	
No increase	178 (39.7%)
Increase > 10%	224 (50%)
Increase > 25%	113 (25.2%)
Shift	
1 (day)	385 (85.9%)
2 (night)	63 (14.1%)
Venous access site (temporary catheter)	
Femoral veins	244 (54.5%)
Right internal jugular vein	135 (30.1%)
Left internal jugular vein	63 (14.1%)
Subclavian veins	1 (0.2%)
**Laboratory blood tests (on dialysis day)**	
Hemoglobin (g/dL)	7.8 (7.0–8.6)
Platelets (1,000/mm3)	169 (91–269)
INR	1.23 (1.1–1.45)
APTT (ratio)	1.22 (1.06–1.44)
CRP (mg/L)	110 (54.8–202)
Lactate (mg/dL)	13 (10–18)
**Anticoagulation**	
Non-fractionated heparin	134 (29.9%)
Saline flush (continuous)	314 (70.1%)
Contraindication for heparin use	
Thrombocytopenia*	183 (41.3%)
Surgical or invasive procedures**	84 (18.7%)
Bleeding (active or occult)***	74 (16.5%)
Coagulopathy†	52 (11.6%)

Results are expressed in number (%) or median (25–75 IQR). IQR, interquartile range; UF, ultrafiltration; INR, international normalized ratio; APTT, activated partial thromboplastin time; CRP, C reactive protein

*thrombocytopenia, platelets < 100,000/mm3

**pre or post surgery, catheter insertion, biopsy

***occult bleeding, hemoglobin decrease without bleeding exteriorization;

†coagulopathy, INR and/or APTT ratio over 2.0.

### Complications of IHD

There were no complications in 316 (70.5%) of the sessions. The most frequent complications were hypotension and clotting. The occurrence of serious complications was low: arrhythmias were observed in three sessions (0.7%); seizures were observed in other three sessions (0.7%). One of the patients presenting seizure required mechanical ventilation. One patient (0.1%) presented a stroke in one session and died a few days later. This death was considered as probably directly related to the procedure. No major bleeding related to heparin was observed.

#### Hypotension

When initiating each IHD session (considering sequential procedures after first session), 18.1% of the patients were on vasoactive drugs: noradrenaline in 13.2% [dose 0.10 (0.06–0.15) mcg/Kg/min] and dobutamine in 4.9% [dose 2.73 (2.44–4.26) mcg/Kg/min]. The initial (iMAP) and minimal MAPs (minMAP) during IHD considering all assessed patients were 87.0 (78.3–98.6) mmHg and 75.3 (64.0–87.5) mmHg, respectively. In patients with hypotension, iMAP and minMAP were 81.0 (71.5–85.7) mmHg and 59.3 (52.3–61.3) mmHg, respectively. A total of 98% of patients presenting hypotension had a blood pressure reduction (initial MAP to minimum MAP) over 10% and 85% of patients over 20%. Hypotension occurred in 73 (49%, 73/149) of patients and 57 (78.1%, 57/73) patients presented hypotension in only one IHD session. Patients who presented hypotension received 2.5 (1.0–3.5) IHD, which was not different from the number of procedures of patients who do not experienced hypotension (*P* = 0.305). Hypotension during IHD occurred in 25% (118/448) of the treatments, causing dialysis interruption in 9.8% (44/448) of them. The hypotension-related interventions were introduction and/or increase in vasopressors in 78% (93/118) of these sessions, UF rate reduction in 28% (33/118) and fluid infusion in 14.4% (17/118). More than one hypotension-related intervention was performed in 25 procedures.

Patients with IHD-associated hypotension (n = 118) presented a higher SOFA score (without renal points) (*P* < 0.001), lower initial MAP (*P* < 0.001) and higher lactate level (*P* < 0.001). Hypotension was also associated with lower dialysate conductivity (*P* < 0.001) and lower achieved ultrafiltration (*P* < 0.001). Hypotension was more frequent in patients with sepsis (*P* = 0.003), those using noradrenaline (*P* = 0.02) and those who received chemotherapy (*P* = 0.02). Dialysate temperature was similar between the groups with and without hypotension (*P* = 0.50) (Table 3).

**Table 3:** Comparison of the groups with and without hypotension during intermittent hemodialysis.

	Without Hypotension	With Hypotension	
Characteristics	(n = 330)	(n = 118)	*P* Value ^§^
Age (years)	59.6 ± 15.0	60.0 ± 16.4	0.40
Primary cancer			
Genitourinary	150 (45.5)	33 (28.2)	0.001
Gastrointestinal	42 (12.7)	14 (12.0)	0.80
Lymphoma/leukemia	30 (9.1)	16 (13.7)	0.16
Myeloma	22 (6.7)	11 (9.4)	0.33
Metastatic tumor	118 (37.6)	34 (31.8)	0.28
Chemotherapy*	54 (16.4)	30 (26.1)	0.02
Cancer status			
No tumor evidence	27 (8.2)	18 (15.2)	0.07
Controlled	47 (14.2)	11 (9.3)	0.18
Uncontrolled newly diagnosed/WT	119 (36.1)	26 (22.0)	0.006
Uncontrolled recurrence/progression	115 (34.8)	52 (44.1)	0.07
Baseline serum creatinine	1.14 (0.90–1.93)	1.02 (0.88–1.30)	0.13
Diabetes mellitus	93 (28.3)	36 (31.3)	0.54
Heart failure	34 (10.3)	11 (9.6)	0.81
Sepsis	131 (39.8)	64 (55.7)	0.003
Surgery	82 (24.9)	21 (18.3)	0.14
Shift 1 (vs. 2)	48 (14.5)	15 (12.8)	0.64
Dialysis duration (minutes)	240 (180–300)	240 (180–325)	0.78
Blood/dialysate flow (ml/min)	250 (200–300)	250 (200–300)	0.56
Achieved ultrafiltration (ml/min)	2.77 (0.0–5.5)	5.50 (0.8–8.30)	<0.001
Dialysate temperature (°C)	37.1 (36.9–37.3)	37.0 (36.8–37.2)	0.56
Dialysate conductivity (ms/cm)	139 (138–140)	138 (137–139)	0.003
Urinary output (mL/24 h)	285 (15.0–980)	178 (0.0–731)	0.050
MAP (initial)	91.5 (81.0–102)	81.0 (71.5–85.6)	<0.001
Initial MAP < 90 mmHg	151 (45.8)	99 (84.6)	<0.001
Noradrenaline **	36 (10.9)	23 (19.7)	0.016
Noradrenaline dose (mcg/Kg/min) **	0.09 (0.06–0.12)	0.11 (0.07–0.20)	0.18
Mechanical ventilation	36 (10.9)	21 (17.9)	0.050
SOFA (without renal points)	3.0 (2.0–5.0)	4.0 (3.0–6.0)	<0.001
Serum lactate (mmol/L)†	12.0 (9.0–17.0)	15.0 (10.0–20.0)	<0.001
Serum CRP†	108 (54.8–184)	123 (50–249)	0.12
Serum hemoglobin (g/dL)†	7.8 (7.0–8.6)	7.7 (7.0–8.6)	0.79
Serum sodium (mEq/L)‡	137 (132–142)	137 (135–143)	0.20
Serum ionized calcium (mg/dL) ‡	4.50 (4.30–4.80)	4.60 (4.30–4.70)	0.57
Serum bicarbonate (mEq/L) ‡	20.6 (19.1–22.4)	20.3 (19.2–22.7)	0.98

Results are expressed in number (%), mean ± SD or median (25–75 IQR). SD, standard deviation; IQR, interquartile range; WT, waiting for treatment; UF, ultrafiltration; MAP, mean arterial pressure; SOFA, sequential organ failure assessment; CRP, C reactive protein.

^§^Analysis was performed based on terms of comparative groups (with and without outcomes) and not compared based on repeated measure analysis.

*In the last four weeks

** At the moment of session initiation.

†Routine daily exams.

‡Pre session blood tests.

The variables independently associated with hypotension were dialysate conductivity, initial MAP and SOFA score (without renal points). Aiming to reduce possible bias from patients’ characteristics that could be sequentially repeated in patients performing a large number of sessions, an additional model was developed with patients and sessions characteristics observed on the first two sessions (n = 235). The variables retained on the model were also dialysate conductivity, initial MAP and SOFA score (without renal points) (Table 4).

**Table 4:** Logistic regression model for hypotension during intermittent hemodialysis. Model A: simple model; Model B: mixed effect for repeated measures model.

	*P*	OR (95% CI)
Model A (simple model)*		
Dialysate conductivity (ms/cm)	0.008	0.81 (0.69–0.95)
Initial MAP (each 10 mmHg)	< 0.001	0.49 (0.40–0.61)
SOFA score (without renal points)	0.003	1.14 (1.02–1.28)
**Model B (mixed effect for repeated measures model)** * *		
Dialysate conductivity (ms/cm)	0.023	0.85 (0.74–0.97)
Initial MAP (each 10 mmHg)	< 0.001	0.50 (0.40–0.63)
SOFA score (without renal points)	0.003	1.19 (1.06–1.33)

* Model performance: area under ROC curve = 0.77 (0.72–0.81); Hosmer-Lemeshow χ2 = 0.82.

** Model performance: area under ROC curve = 0.76 (0.71–0.81)

ROC, receiver operating characteristic; OR, odds ratio; CI, confidence interval; MAP, mea arterial pressure; SOFA, sequential organ failure assessment.

#### System clotting and malfunctioning catheter (MC)

MC was observed in 29.2% (131/448) of the sessions, lines reversion in 23.7% (106/448) and blood flow reduction in 8.9% (40/448). Both MC and blood flow reduction were observed in 3.3% (15/448) of the procedures. Clotting leading to IHD interruption occurred in 23.8% of the procedures. Clotting (n = 107) was more frequently observed in the presence of MC (*P* < 0.001), system pressure increase (SPI) over 25% (*P* < 0.001), dialysis duration ≥ 5 h (*P* < 0.001), myeloma (*P* = 0.009), night shift (*P* = 0.03) jugular vein catheter site (*P* = 0.005) and less frequently observed in the case of heparin use (*P* = 0.03), platelets < 100,000/mm3 (*P* = 0.004), sepsis (*P* = 0.03), chemotherapy (*P* = 0.02), uncontrolled tumor disease (*P* = 0.04), gastrointestinal tumor (*P* = 0.03) and femoral vein catheter site (*P* = 0.01) (Table 5). The independent risk factors for clotting were heparin use, MC and SPI over 25%. An additional model was also developed with patients and sessions characteristics of the first two sessions (n = 235) and the variables retained in the model were MC, SPI over 25% and platelets < 100,000/mm3 (Table 6).

**Table 5:** Comparison of the groups with and without clotting during intermittent hemodialysis.

	Without Clotting	With Clotting	
Characteristics	(n = 341)	(n = 107)	*P* Value ^§^
Age (years)	59.4 ± 16.1	60.1 ± 16.0	0.51
Primary cancer			
Genitourinary	175 (51.3)	65 (60.7)	0.09
Gastrointestinal	49 (14.4)	7 (6.5)	0.03
Lymphoma/leukemia	38 (11.1)	8 (7.5)	0.28
Myeloma	19 (5.6)	14 (13.1)	0.009
Metastatic tumor (for solid cancer)	113 (34.8)	40 (41.2)	0.24
Chemotherapy*	73 (21.5)	12 (11.3)	0.02
Cancer status			
No tumor evidence	31 (9.1%)	14 (12.1)	0.20
Controlled	48 (14.1)	10 (9.3)	0.20
Uncontrolled newly diagnosed/WT	103 (30.2)	42 (39.3)	0.08
Uncontrolled recurrence/progression	137 (40.2)	31 (29.0)	0.04
Baseline SCr (mg/dL)	1.15 (0.89–1.93)	1.0 (0.89–1.30)	0.24
Sepsis	159 (46.9)	37 (34.9)	0.03
Night shift (vs. day shift)	41 (12.0)	22 (20.6)	0.03
Dialysis duration (minutes)	240 (180–300)	240 (180–320)	0.4
Dialysis duration ≥ 5 h	100 (29.3)	53 (49.5)	<0.001
Blood/dialysate flow (mL/min)	250 (200–300)	250 (200–300)	0.33
Prescribed UF (mL/min)	5.55 (1.87–8.33)	4.16 (0.0–6.94)	0.03
System pressure increase (over 25%)	90 (26.8)	52 (49.1)	<0.001
Catheter site			
Jugular vein	138 (40.5)	60 (56.1)	0.005
Right jugular vein	92 (27.0)	43 (40.2)	0.009
Left jugular vein	46 (13.5)	17 (15.9)	0.53
Femoral vein	197 (57.8)	47 (43.9)	0.012
Heparin	111 (32.6)	23 (21.5)	0.03
Malfunctioning catheter	75 (22.0)	56 (52.3)	< 0.001
SOFA (without renal points)	4.0 (2.0–6.0)	4.0 (2.0–6.0)	0.15
MAP (initial, mmHg)	88.6 (78.3–98.8)	85.3 (78–96)	0.16
Hypotension	92 (27.1)	25 (23.4)	0.448
Serum lactate (mmol/L)	13 (10–18)	13 (9–17)	0.75
Serum CRP (mg/L)	114 (54.8–205)	92 (52.1–197)	0.33
Serum hemoglobin (g/dL)	7.8 (7.1–8.6)	7.8 (7.0–8.5)	0.82
Serum platelets (1,000/mm3)	172 (72–271)	167 (135–261)	0.20
Thrombocytopenia**	102 (30.3)	17 (16.0)	0.004
INR	1.24 (1.10–1.48)	1.22 (1.16–1.31)	0.88
APTT (ratio)	1.25 (1.04–1.50)	1.22 (1.16–1.31)	0.10

Results are expressed in number (%), mean ± SD or median (25–75 IQR).

^§^Analysis was performed based on terms of comparative groups (with and without outcomes) and not compared based on repeated measure analysis. SD, standard deviation; IQR, interquartile range

*In the last four weeks; WT, waiting for treatment; SCr, serum creatinine; UF, ultrafiltration; SOFA, sequential organ failure assessment; MAP, mean arterial pressure; CRP, C reactive protein

**thrombocytopenia, platelets < 100,000/mm3; INR, international normalized ratio; APTT, activated partial thromboplastin time.

**Table 6:** Logistic regression model for clotting during intermittent hemodialysis. Model A: simple model; Model B: mixed effect for repeated measures model.

Variable	*P*	OR (95% CI)
Model A (simple)*		
Heparin (yes/no)	0.047	0.57 (0.33–0.98)
Malfunctioning catheter	< 0.0001	3.59 (2.24–5.77)
System pressure increase over 25%	< 0.0001	2.59 (1.61–4.17)
Model B (mixed effect for repeated measures model)* *		
Platelets < 100,000/mm3	0.047	0.28 (0.08–0.98)
Heparin (yes/no)	0.032	0.48 (0.25–0.94)
Malfunctioning catheter	< 0.0001	2.92 (1.77–4.80)
System pressure increase over 25%	0.001	2.17 (1.40–3.38)

* Model performance: area under ROC curve = 0.71 (0.65–0.77); Hosmer-Lemeshow χ2 = 0.76.

** Model performance: area under ROC curve = 0.72 (0.66–0.77); ROC, receiver operating characteristic; OR, odds ratio; CI, confidence interval.

#### Adequacy

Pre and post IHD blood tests were available in 313 (69.8%) sessions. IHD provided an adequate metabolic control, and blood test parameters were significantly improved after RRT completion: urea decreased from 161 (111–216) to 71 (49–104) mg/dL (*P* < 0.001); SCr from 5.66 (4.35–7.80) to 2.71 (2.10–3.80) mg/dL (*P* < 0.001); serum potassium from 4.5 (3.7–5.1) to 3.8 (3.5–4.1) mEq/L (*P* < 0.001); phosphate from 5.3 (4.3–7.0) to 2.8 (2.1–3.7) mg/dL (*P* < 0.001) and bicarbonate increased from 20.5 (19.1–22.4) to 24.1 (22.5–25.5) mEq/L (*P* < 0.001). The delivered Kt/V per session was 0.87 (0.64–1.15), and the URR was 53.3 (43.5–62.8)%.

Occurrence of clotting reduced efficiency when procedures with and without clotting were compared: Kt/V 0.71 [0.56–0.93] versus 0.94 [0.69–1.18]), *P* < 0.001; and URR 45.6 [38.4–56.6] % versus 56.2 [46.6–64.4] %, *P* < 0.001. Clotting was also associated with reduced delivered UF: 4.1 [0.0–6.2] versus 4.8 [0.0–8.3] ml/min, ***P =*** 0.05. Heparin use and malfunctioning catheter did not have an impact on either urea kinetics or UF (data not shown). Hypotension was associated with a reduced delivered UF (2.77 [0.0–5.5] vs. 5.5 [0.8–8.3] ml/min, *P* < 0.001) but did not impact Kt/V (*P* = 0.70) or URR (*P* = 0.70).

## Discussion

There are an increasing number of cancer patients with AKI who receive RRT in the ICUs worldwide. In this observational, prospective study, we evaluated a large number of IHD procedures performed in a sizable group of critically ill cancer patients with AKI. To the best of our knowledge, this study is the first to describe in detail technical characteristics of IHD in this group of patients. IHD is currently used in the critical care setting in hemodynamically stable patients [13, 14]. We were able to use IHD in 55.2% of the cancer patients with AKI in the ICU who received RRT, and 34.1% of these patients received exclusively IHD during their ICU stay. We demonstrated that IHD complications rates were not greater to that reported for non-cancer patients with AKI in the ICU [25, 26].

In the current study, over 70% of procedures completed both the prescribed UF and duration. IHD therapy provided satisfactory metabolic control, and blood test parameters significantly improved after RRT completion. RRT is a key aspect in the care of ICU patients, and the valuable information offered by the present manuscript could help to plan a RRT prescription tailored to the patients´ characteristics, avoid major complications, improve metabolic control and also optimize costs. Moreover, different from previous studies, our data provided important data on solid tumor cases that are frequently observed in general ICUs worldwide.

Darmon M. *et al*. [5] and Maccariello E. *et al*.[6] prospectively evaluated cancer patients with AKI in the ICU who received RRT, aiming to assess prognostic factors. The former survey (n = 94) was comprised of mostly hematology malignancy (77.7%) and the second (n = 118) was comprised of mostly solid tumor malignancy (73%). In both surveys, most patients were on mechanical ventilation and vasopressors. Hospital mortality was 51.1 and 78%, respectively. Both series demonstrated that cancer patients´ mortality was similar to that reported for non- cancer patients, but the authors did not offer data on complications or dialysis efficiency.

Salahudeen A.K. *et al*. [12] retrospectively assessed 199 ICU cancer patients requiring RRT. Most patients (62%) had hematology neoplasia, sepsis was present in 27% of the cases and 30 day mortality was 65%. All patients received “continuous” SLED (sustained low-efficiency extended dialysis). Although 75% of the patients were on vasopressors before dialysis initiation, a satisfactory UF was achieved, associated with satisfactory hemodynamic stability. This study was the first to describe the technical characteristics of RRT in cancer patients in the ICU, but was performed mainly in hematologic cancer patients and was retrospective.

In the current study, there were no complications in 70.5% of the IHD sessions, a serious event occurred in 1.7% of the sessions, and only one death was considered to be related to IHD [27]. Hypotension was observed in 25% of the sessions. This rate is similar to that reported in previous studies assessing SLED in AKI ICU non cancer patients, but the currently studied patients were less severely ill than those included in these non-cancer series [28, 29]. SPB does not allow a dialysate sodium profile or temperature control, which could be a drawback for avoiding hypotension. Conversely, SPB might reduce hypotensive episodes due to spontaneous cooling of dialysate (0.3° C per hour) and high bacteriological quality (ultrapure water)[30, 31].

Clotting leading to IHD interruption occurred more frequently in the case of MC, no heparin use and SPI over 25%. The first two factors have been already reported as important for clotting in SLED and IHD-treated AKI patients [32]. It could be speculated that SPI might be the cause or consequence of clotting. However, in the present study, SPI preceded clotting and was not influenced by important clotting-related factors (heparin and MC).

Patients with some types of solid cancer (breast, prostate, head and neck, gastric, pancreatic, sarcomas), present increased levels of pro-inflammatory cytokines such as interleukin 1, interleukin 6, tumor necrosis factor α, which are associated to systemic effects, such as cachexia, pain, toxicity and resistance of treatment [33, 34]. In addition, cancer patients have a pro-thrombotic switch of the hemostatic system, which has a complex pathogenesis, including dysfunction of endothelial cells, tumor cells expression of adhesion molecules that bind platelets, endothelial cells and leucocytes and increased production of different procoagulant proteins (tissue factor that generates thrombin, factor VII), inflammatory cytokines (IL-1, TNF-alfa), and procoagulant microparticles that might trigger clotting [35, 36]. Both pro-thrombotic and pro-inflammatory status are enhanced in the presence of advanced cancer disease as well as after cancer treatments such as chemo and radiotherapy [33–36]. In the same way, blood-dialyzer interaction during hemodialysis activates mononuclear cells leading to the production of inflammatory cytokines and clotting pathways [37]. Therefore, RRT initiation in patients already in a cancer related pro-inflammatory and pro-thrombotic status might increase these patients vulnerability to hemodynamic instability and filter clotting. We emphasize that approximately 70% of our patients have uncontrolled cancer disease, 36.2% of them with metastases to distant organs. Herein, we suggest that cancer patients must be considered as a group with particular characteristics and so, nephrologists should evaluate cancer status and recent cancer treatments, in order to add these aspects to the parameters routinely used when prescribing RRT.

We observed that the characteristics of our patients were similar to those reported in previous studies regarding demographic aspects (age, gender comorbidities) and hospital mortality (59.7%). Conversely, our patients were less severely ill compared to other surveys because only up to 10% of them required mechanical ventilation and vasopressors, and SOFA scores were relatively low when initiating IHD [5, 6, 12]. Although sepsis was observed in 51% of the patients, obstructive uropathy and surgery were associated with AKI in 27.5 and 24.2% of patients, respectively. Of note, patients in the present series presented a high tumor burden, with almost 80% with uncontrolled cancer disease and 30% with metastatic disease. It should be stressed that more critically ill and hemodynamically unstable patients in our ICU undergo CRRT. So, our IHD patients are not representative of the whole ICU population. Around 30% of patients treated at ICESP ICU required mechanical ventilation and vasopressors in a series reported by Hajjar LA et al [38].

Our study has some limitations. It is a single center study, performed in an academic, tertiary hospital, exclusively devoted to the care of cancer patients. RRT was handled by a specialized nursing team. The studied patients were less severely ill than those usually described in previous reports, and it is likely that most unstable and frail or at risk of deteriorating patients were selected for CRRT. We cannot exclude possible other selection biases due to local specificities related to the standards of care and criteria to initiate RRT. All of these aspects could make our data less generalizable. Finally, we have no data on fluid balance, nutrition status and cancer related patient´s performance.

## Conclusions

In summary, IHD was possible to perform in a significant group of critically ill cancer patients requiring RRT due to AKI. IHD offered no extra risk to the patient suffering with cancer and AKI as a treatment when compared with other outcome data of IHD in AKI in non-cancer patients. IHD offered reasonable hemodynamic stability and provided satisfactory metabolic control. These data indicate that IHD might be offered as therapeutic modality for hemodynamically stable cancer patients with AKI in the ICU.
